# The nervous system of the most complex lophophore provides new insights into the evolution of Brachiopoda

**DOI:** 10.1038/s41598-021-95584-5

**Published:** 2021-08-10

**Authors:** Elena N. Temereva, Tatyana V. Kuzmina

**Affiliations:** 1 Department Invertebrate Zoology, Biological Faculty, Moscow State University, Leninskie Gory, 1-12, Moscow, Russia 119991; 2 Faculty of Biology and Biotechnology, National Research University Higher School of Economics, Moscow, Russia

**Keywords:** Evolution, Zoology

## Abstract

The lophophore is a tentacle organ unique to the lophophorates. Recent research has revealed that the organization of the nervous and muscular systems of the lophophore is similar in phoronids, brachiopods, and bryozoans. At the same time, the evolution of the lophophore in certain lophophorates is still being debated. Innervation of the adult lophophore has been studied by immunocytochemistry and confocal laser scanning microscopy for only two brachiopod species belonging to two subphyla: Linguliformea and Rhynchonelliformea. Species from both groups have the spirolophe, which is the most common type of the lophophore among brachiopods. In this study, we used transmission electron microscopy, immunocytochemistry, and confocal laser scanning microscopy to describe the innervation of the most complex lophophore (the plectolophe) of the rhynchonelliform species *Coptothyris grayi*. The *C. grayi* lophophore (the plectolophe) is innervated by three brachial nerves: the main, second accessory, and lower. Thus, the plectolophe lacks the accessory brachial nerve, which is typically present in other studied brachiopods. All *C. grayi* brachial nerves contain two types of perikarya. Because the accessory nerve is absent, the cross nerves, which pass into the connective tissue, have a complex morphology: each nerve consists of two ascending and one descending branches. The outer and inner tentacles are innervated by several groups of neurite bundles: one frontal, two lateral, two abfrontal, and two latero-abfrontal (the latter is present in only the outer tentacles). Tentacle nerves originate from the second accessory and lower brachial nerves. The inner and outer tentacles are also innervated by numerous peritoneal neurites, which exhibit acetylated alpha-tubulin-like immunoreactivity. The nervous system of the lophophore of *C. grayi* manifests several evolutionary trends. On the one hand, it has undergone simplification, i.e., the absence of the accessory brachial nerve, which is apparently correlated with a reduction in the complexity of the lophophore’s musculature. On the other hand, *C. grayi* has a prominent second accessory nerve, which contains large groups of frontal perikarya, and also has additional nerves extending from the both ganglia to the medial arm; these features are consistent with the complex morphology of the *C.* *grayi* plectolophe. In brachiopods, the evolution of the lophophore nervous system apparently involved two main modifications. The first modification was the appearance and further strengthening of the second accessory brachial nerve, which apparently arose because of the formation of a double row of tentacles instead of the single row of the brachiopod ancestor. The second modification was the partial or complete reduction of some brachial nerves, which was correlated with the reduced complexity of the lophophore musculature and the appearance of skeletal structures that support the lophophore.

## Introduction

Brachiopods are sessile benthic marine animals that have a bivalve shell. This phylum appeared in the early Cambrian and was dominant in many past marine communities^1,2^. Brachiopod species were very abundant in the past, but there are only about 400 species in recent fauna^3^. In bilaterian phylogeny, brachiopods together with phoronids and bryozoans are grouped into the Lophophorata clade. The lophophorates monophyly has been recently confirmed based on both morphological^4–8^ and molecular data^9,10^.

Like all other lophophorates, brachiopods have a tentacular organ, the lophophore, which collects food particles from the water column^11,12^. The lophophore consists of a brachial axis, which is a ribbon-like structure bearing a row of tentacles. Anteriorly, the brachial axis always forms an open loop, and the rudiments of new tentacles form at its end^13^. The brachial fold stretches along the tentacles, and the brachial groove is located between the brachial fold and tentacles^14^. The brachial axis can twist in different directions and generally determines the morphology of the lophophore. Recent brachiopods exhibit nine types of lophophore morphology^15^. The morphologically simplest lophophores are the taxolophe (occurring only in ontogenesis) and the trocholophe, and the most complex is the plectolophe.

Traditionally, the evolution of the brachiopod lophophore is thought to have involved increasing complexity, i.e., the lophophore was thought to have evolved from the simple ring-like structure of the trocholophe to a very complex plectolophe with three arms^14–16^. Detailed analyses of the literature including descriptions of extinct species revealed that the simple spirolophe rather than the trocholophe is the ancestral type of the lophophore in brachiopods^1,13^. How the simple spirolophe can give rise to simpler forms, such as the trocholophe and schizolophe, or to more complex forms, such as the ptycholophe or plectolophe, is still unclear. It was recently assumed that paedomorphosis played a substantial role in the formation of different types of lophophores^13^. The same pathway, i.e., paedomorphosis, has recently been suggested to explain the evolution of the lophophore in phoronids. According to this view, the phoronid lophophore evolved in two different ways: simplification from an ancestral horseshoe-shed type to an oval lophophore via paedomorphosis and complication from ancestral horseshoe-shed type to spiral lophophore^17,18^.

The phylum Brachiopoda contains three subphyla: Linguliformea, Craniiformea, and Rhynchonelliformea. The relationship between these subphyla is also undefined^19–21^. The brachiopod lophophore has been infrequently studied by immunocytochemistry and confocal laser scanning microscopy (CLSM): only two adult species have been studied by these methods—*Lingula anatina* (from Linguliformea)^4^ and *Hemithiris psittacea* (from Rhynchonelliformea)^22^. Both species have a spirolophous lophophore (the spirolophe). The lophophore nervous system has also been described in *Novocrania anomala* juveniles, which have morphologically simple lophophores, i.e., trocholophe and schizolophe^23^.

Based on recent reports, researchers have noted that, for lophophorates in general and for brachiopods in particular, the organization of the lophophore nervous system is closely related to the morphology of the lophophore, i.e., morphologically simple lophophores have many nerve tracts, and morphologically complex lophophores have only a few nerve tracts^18,22^. Thus, in brachiopod with complex spirolophe (*H. psittacea*), the partial reduction of the accessory brachial nerve, which is very prominent in *L. anatina* without comprehensive spirolophe, has been described^22^. The study of the nervous system organization of the most complex lophophore is important to determine whether there is correlation between the lophophore morphology and the nervous system anatomy. Thus, in the current research we studied the innervation of the lophophore of rhynchonelliform *Coptothyris grayi*, a species that has a plectolophe, i.e., a morphologically complex lophophore.

## Results

### Morphology of the lophophore in *C. grayi*

**Figure 1:**
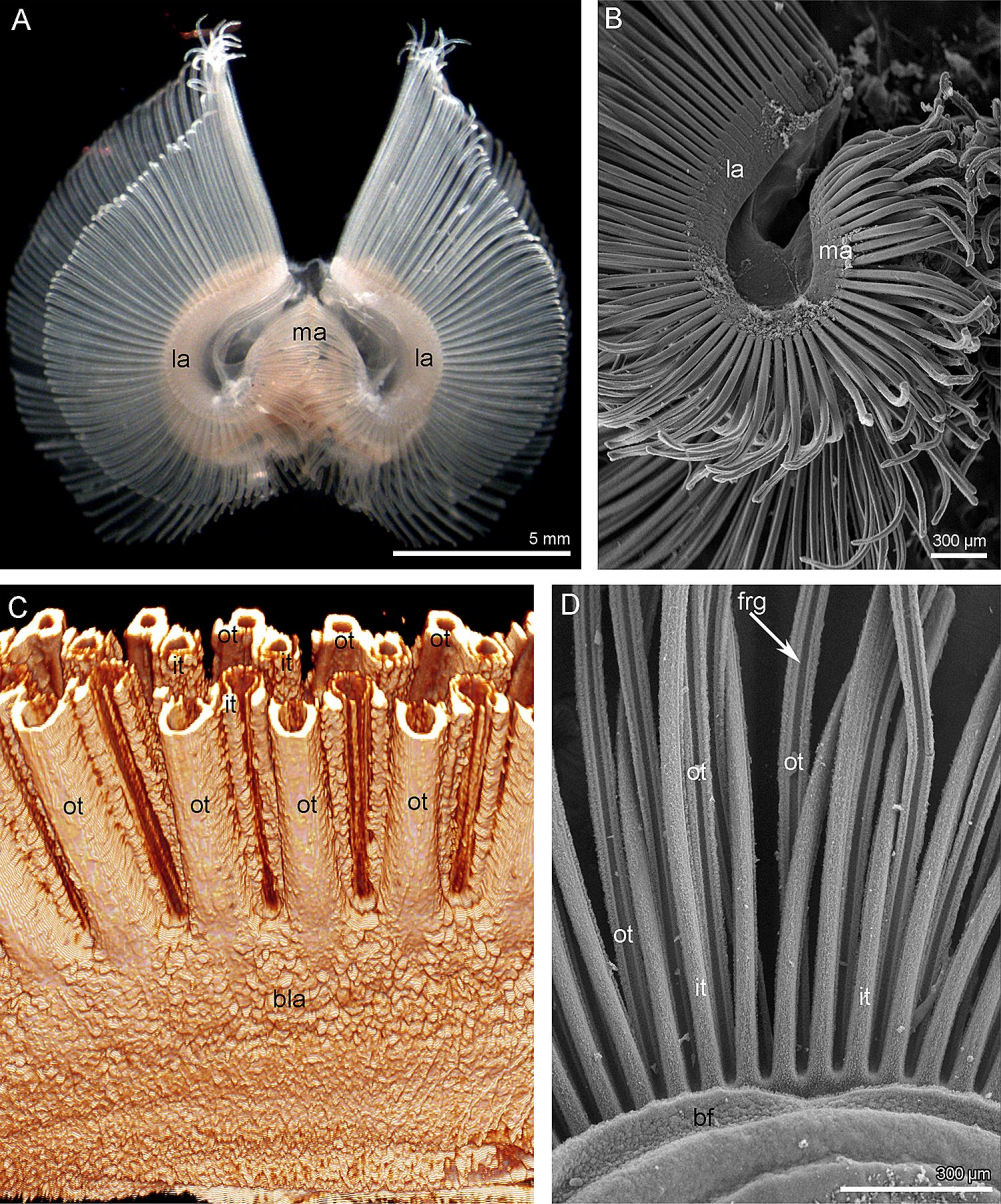
Organization of the lophophore in *Coptothyris grayi*. (**A**) Photograph of narcotized live lophophore. (**B**) A portion of lateral and median arms of the lophophore; SEM. (**C**) A portion of the lateral arm: two double rows of tentacles are visible; volume rendering after immunostaining against acetylated alpha-tubulin; CLSM. (**D**) Organization of the brachial axis, which bears double row of tentacle (inner and outer tentacles) and the brachial fold; SEM. Abbreviations: bf—brachial fold; bla—base of the lateral arm; frg—frontal groove; it—inner tentacle; la—lateral arm; ma—median arm; ot—outer tentacle.

**Figure 2:**
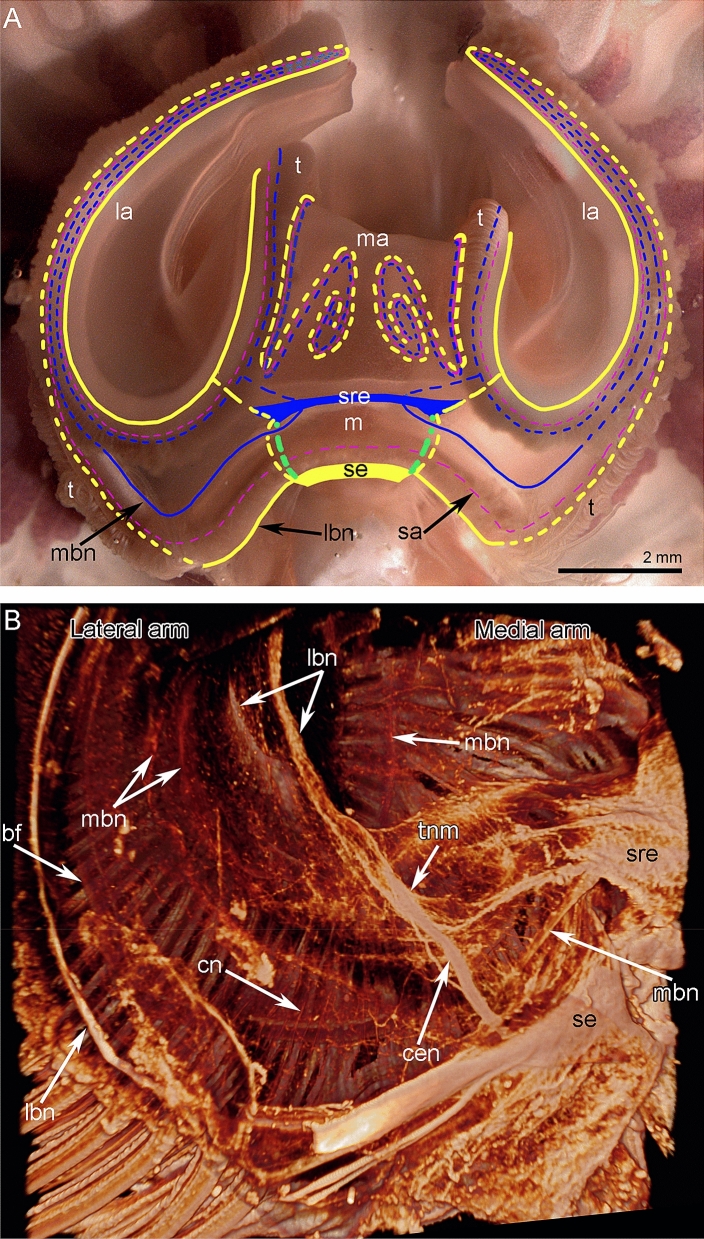
Organization of the central nerous system in *Coptothyris grayi*. (**A**) The photograph of live plectolophe with scheme of location of major nerve elements. Nerves, which are screened by lophophore structures (tentacles and brachial fold), are shown by dotted lines. Solid lines indicate nerves, which extend exactly on the lophophore surface. (**B**) Central portion of the lophophore with parts of lateral and median arms; volume rendering after immunostaining against acetylated alpha-tubulin; CLSM. Abbreviations: bf—brachial fold; cen—circumenteric connective; cn—cross nerve; la—lateral arm; lbn—lower brachial nerve; m—mouth; ma—medial arm; mbn—main brachial nerve; sa—second accessory brachial nerve; se—subenteric ganglion; sre—supraenteric ganglion; t—tentacles; tnm—thick nerves extending to the median arm.

**Figure 3:**
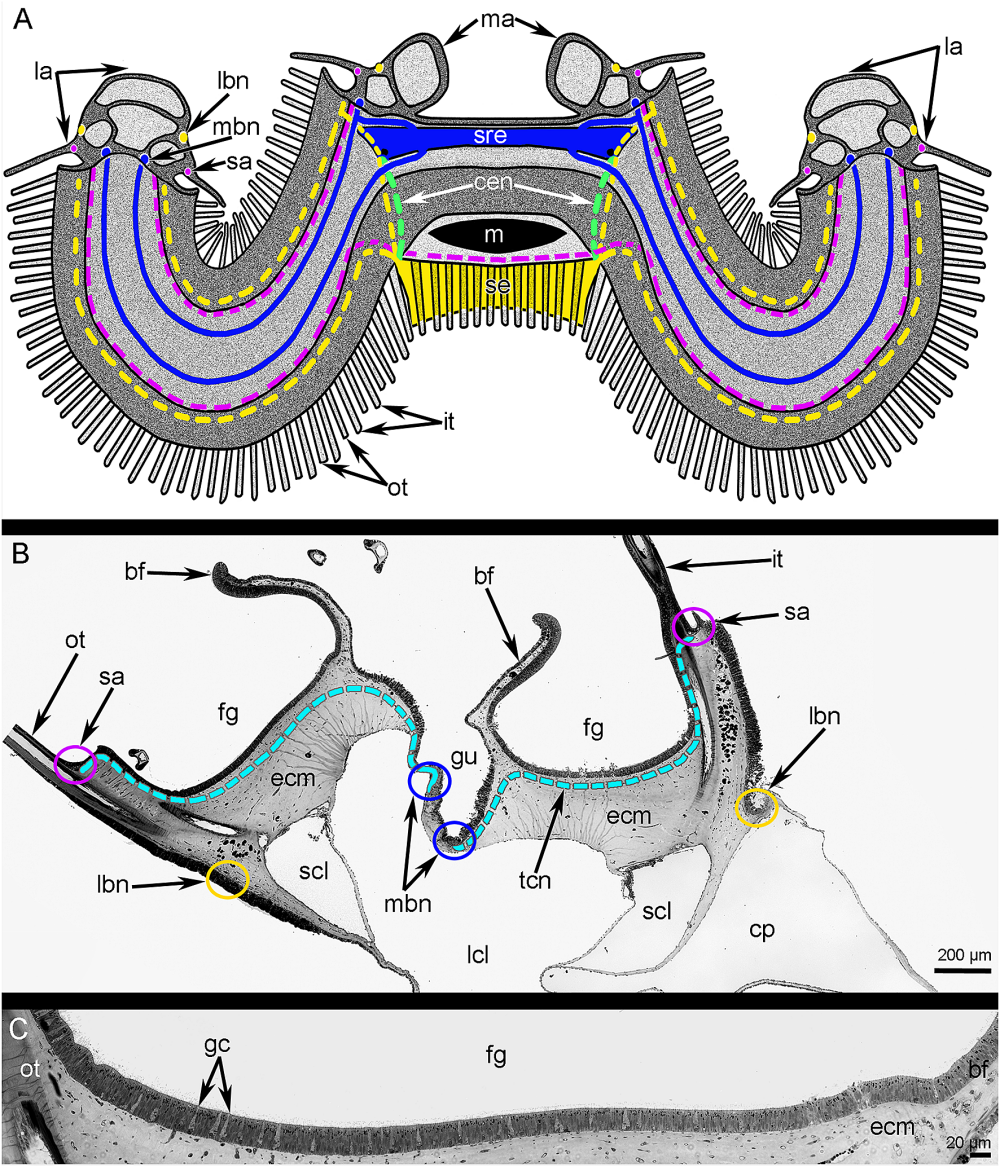
Location of ganglia and brachial nerves in the lophophore of *Coptothyris grayi*. (**A**) The scheme of central part of the lophophore; lateral and median arms are cut. Dotted lines are used to show nerve elements, which are screened by lophophore structures (tentacles and brachial fold). (**B**) Semi-thin cross section of the lateral arm. The location of different nerves is marked by circles of different colors, which correspond to the colors of nerves in Figs. 3A and 6. (**C**) Semithin section of the food grove: the brachial fold is to the right; the tentacles are to the left. Abbreviations: bf—brachial fold; cen—circumenteric nerve; cp—coelomic pouch; ecm—extracellular matrix; fg—food groove; gc—gland cells; gu—gutter; it—inner tentacle; lbn—lower brachial nerve; lcl—large canal of the lophophoral coelom; m—mouth; ma—medial arm; mbn—main brachial nerve; ot—outer tentacle; sa—second accessory brachial nerve; scl—small canal of the lophophoral coelom; se—subenteric ganglion; sre—supraenteric ganglion; tcn—trace of the cross nerves.

*C. grayi* has the most complex type of lophophore among brachiopods—the plectolophe (Fig. 1A). The lophophore consists of three arms: two wing-like lateral arms and one spiral medial arm (Figs. 1A, B, 2A, and 3A). The arms are formed by a strongly curved brachial axis that consists of a double row of tentacles and a brachial fold separated by a food groove. The mouth is located in the middle of the brachial axis in the food groove between the brachial fold and tentacle row. On both sides of the mouth, in the lateral arms, the brachial axis runs along the lower part of each lateral arm, turns backward on its distal end, and passes the upper side of the lateral arm. Above the mouth, the left and right ends of the brachial axis extend into the middle arm, where they form a spiral, and end at the distal end of the middle arm (Fig. 2A). Thus, each arm consists of two brachial axes bearing two rows of tentacles, two brachial folds, and two food grooves (Figs. 1C and 3B). The food groove is flattened and continuous (Fig. 3C). The epithelium of food groove contains numerous gland cells and does not contain prominent nerve elements (Fig. 3C). In the lateral arms, the brachial axes are close to each other, so that the epidermis between the two brachial folds forms a deep brachial gutter (Fig. 3B). Each row of tentacles, except for the area under the mouth, is double and consists alternating inner and outer tentacles: the inner tentacles are closer to the brachial fold than the outer tentacles (Fig. 1C).

**Figure 4:**
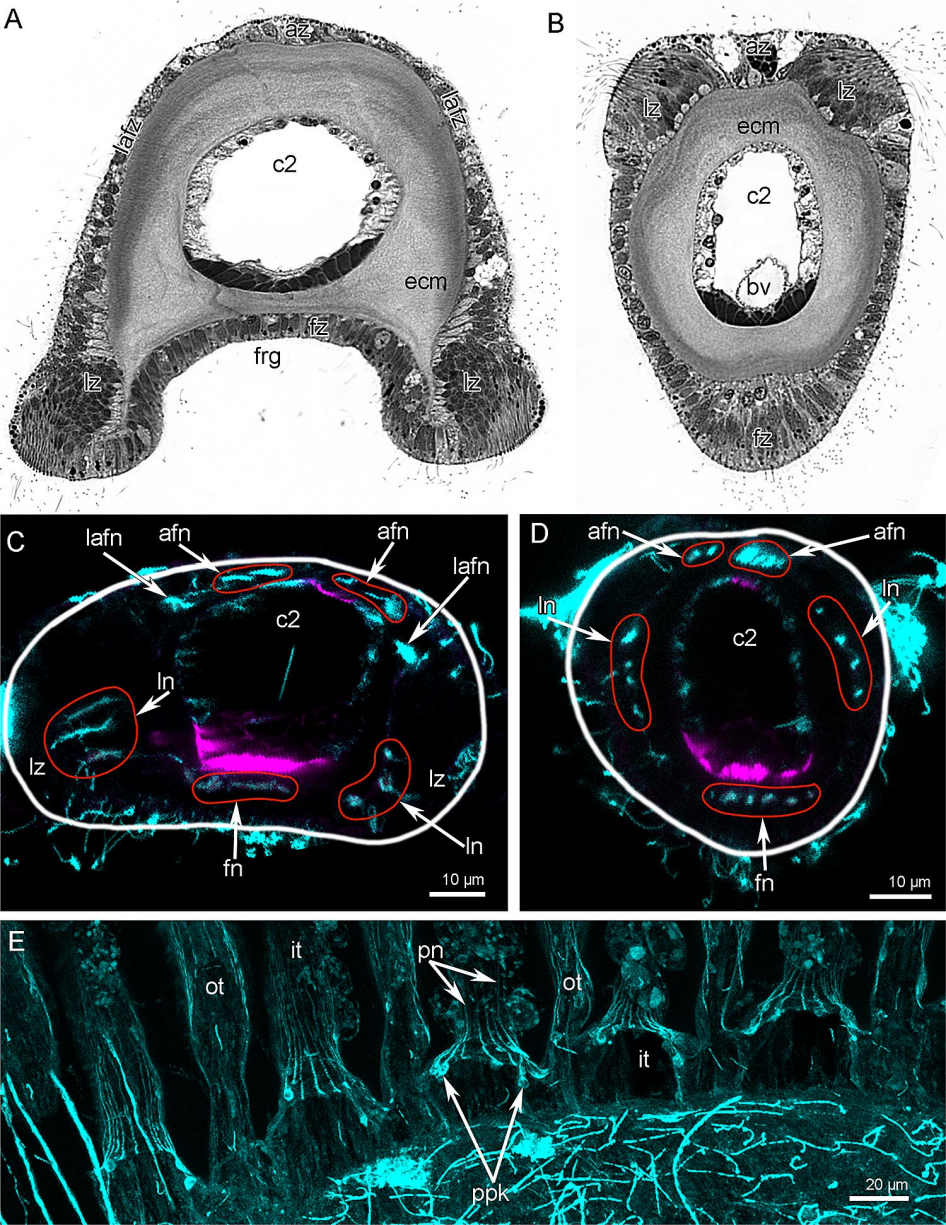
Organization of tentacles in *Coptothyris grayi*. Semi-thin transverse sections (**A**, **B**); Z-projections after immunostaining against acetylated alpha-tubulin (cyan) and staining with phalloidin (magenta) (**C**–**E**). Red circles indicate certain tentacle nerves, which consist of several neurite bundles. (**A**) Outer tentacle. (**B**) Inner tentacle. (**C**) Z-projection of outer tentacle; the border of tentacle is shown by white line. (**D**) Z-projection of inner tentacle; the border of tentacle is shown by white line. (**E**) Z-projection peritoneal neurites in outer and inner tentacles. Abbreviations: afn—abfrontal tentacle nerve; az—abfrontal zone; bv—tentacle blood vessel; c2—tentacle coelom (mesocoel); ecm—extracellular matrix; fz—frontal zone; fn—frontal tentacle nerve; frg—frontal groove; it—inner tentacle; lafn—lateroabfrontal tentacle nerve; lafz—lateroabfrontal zone; ln—lateral tentacle nerve; lz—lateral zone; pn—peritoneal neurite; ot—outer tentacle; ppk—peritoneal perikarya.

**Figure 5:**
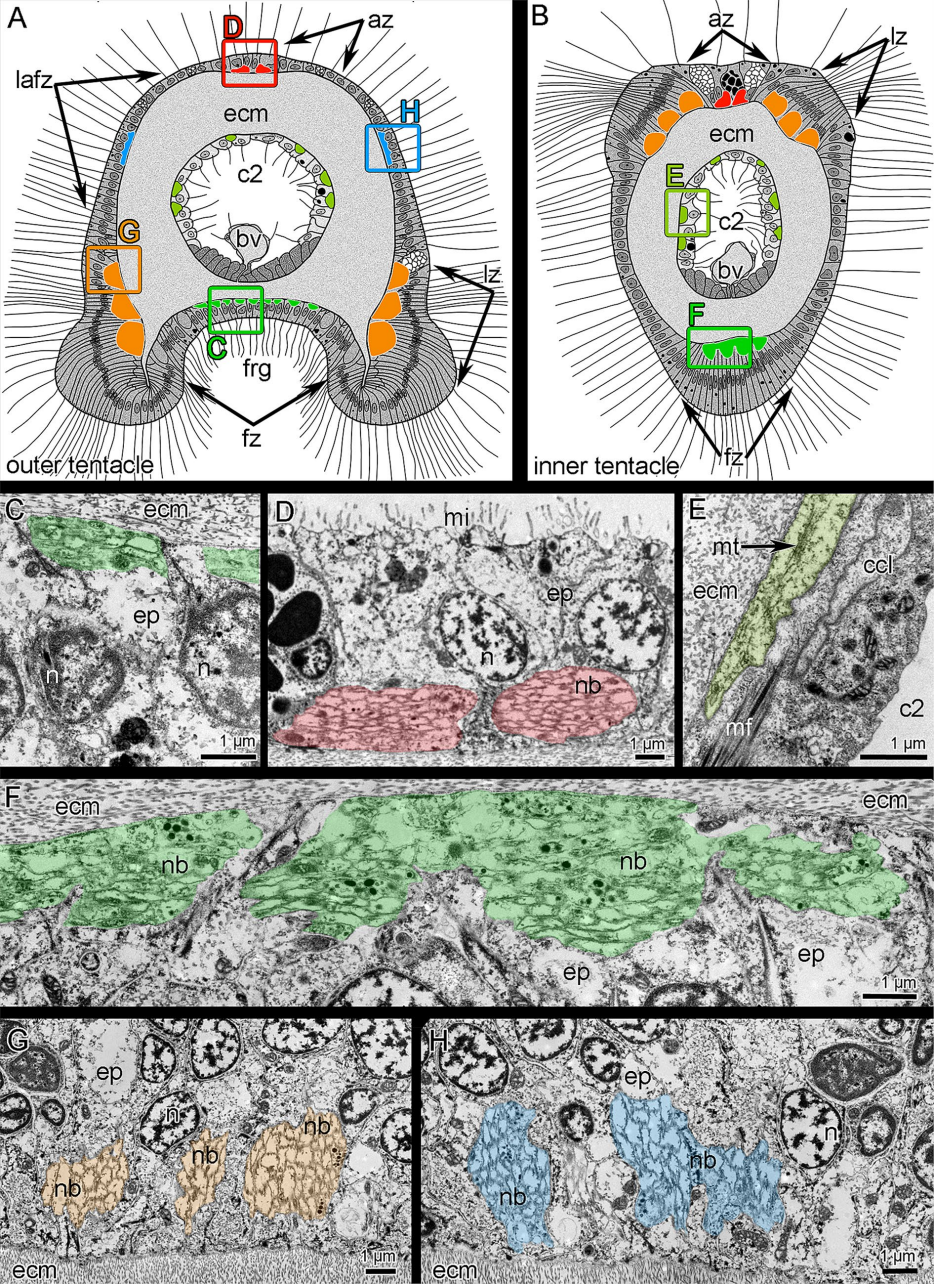
Innervation of tentacles of *Coptothyris grayi*. Schemes of cross section (**A**–**B**)—colored boxes and letters indicate places, which ultrastructure is given in the figure; TEM (**C**–**H**). (**A**) Organization of the outer tentacle. (**B**) Organization of the inner tentacle. (**C**) Frontal neurite bundle of outer tentacle. (**D**) Abfrontal neurite bundle of outer tentacle. (**E**) Peritoneal neurite of inner tentacle. (**F**) A portion of frontal nerve of inner tentacle. (**G**) Lateral tentacle nerve of outer tentacle. (**H**) Lateroabfrontal nerve of outer tentacle. Abbreviations: az—abfrontal zone; bv—tentacle blood vessel; c2—tentacle coelom (mesocoel); ccl—cells of coelomic lining; ecm—extracellular matrix; ep—epithelium; frg—frontal groove; fz—frontal zone; lafz—lateroabfrontal zone; lz—lateral zone; mf—myofilaments; mi—microvilli; mt—microtubule; n—nucleus; nb—neurite bundle.

Both the inner and outer tentacles have zones that differ in ciliation, in position relative to the other tentacles and the brachial fold, and in the histological structure of epithelium (Figs. 1D and 4A, B). Tentacles of both types have eight zones: one frontal, one abfrontal, two latero-frontal, two lateral, and two lateroabfrontal. Inner and outer tentacles differ from each other in form in transverse section and in the location of ciliated zones (Figs. 4A, B and 5A, B). In both inner and outer tentacles, the frontal zone faces the brachial fold. In outer tentacles, the frontal zone is concave and forms a deep frontal groove that is lined with cubic epithelium (Figs. 4A and 5A). In inner tentacles, the frontal side protrudes and bears the ciliated ridge, which is formed by columnar epithelium (Figs. 4B and 5B). The abfrontal zone is opposite to the frontal zone. The abfrontal zone is wide in the outer tentacles but very narrow in the inner tentacles. Lateral zones form two densely ciliated ridges in tentacles of both types. The lateral ciliated ridges are located close to the frontal zone in the outer tentacles and are located close to the abfrontal zone in the inner tentacles. According to these locations of the lateral ridges, there are two latero-abfrontal zones in the outer tentacles, and two extensive latero-frontal zones in the inner tentacles (Fig. 4A, B).

### General anatomy of the nervous system in* C. grayi*

Two ganglia, one subenteric and the other supraenteric, are the main elements of the nervous system in *C. grayi* (Figs. 2A, B and 3A; Suppl. 1). The subenteric ganglion is located under the mouth and gives rise to the lower brachial nerves, which extend into both lateral brachial arms, skirt them, and penetrate into the middle brachial arm (Figs. 2A, B and 3A; Suppl. 1). The supraenteric ganglion is located above the mouth and consists of two lateral nerve centers, which are connected to each other via a thick commissure (Figs. 2A and 3A). The supraenteric ganglion gives rise to the pair of main brachial nerves, which extend at the base of the brachial fold along the middle line of each lateral brachial arm (Fig. 3B). At the end of each lateral arm, each main brachial nerve extends back to the mouth and then penetrates the middle brachial arm. The supraenteric ganglion also gives rise to nerves in the middle brachial arm; these nerves fuse with the main brachial nerves. The subenteric and supraenteric ganglia are connected to each other via thick circumenteric connectives. Near the subenteric ganglion, each connective gives rise to a thick nerve, which extends into the middle brachial arm and joins the lower brachial nerve (Figs. 2B and 3A).

### Innervation of the brachial arms in *C. grayi*

**Figure 6:**
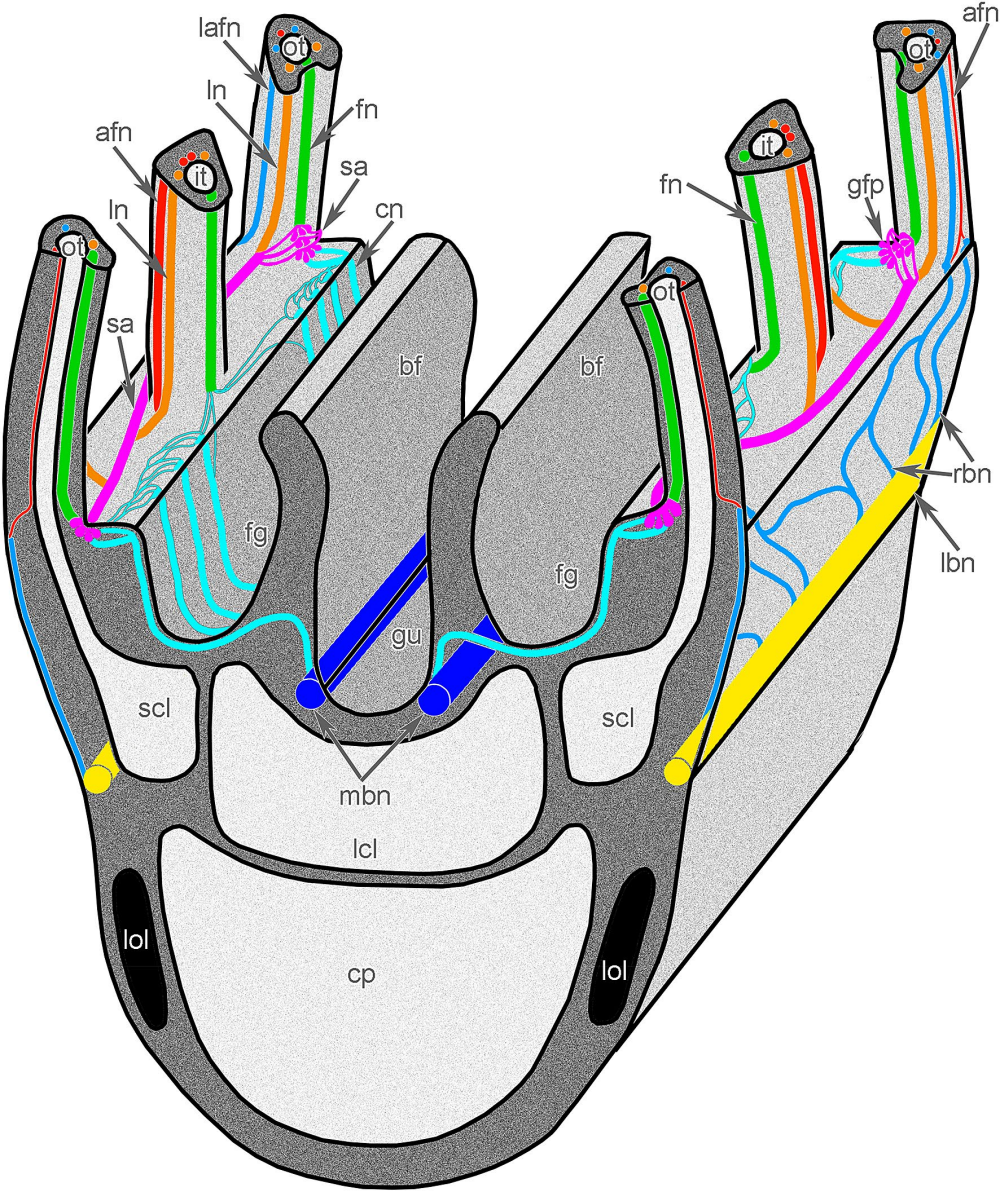
Scheme of innervation of lateral arm of the lophophore of *Coptothyris grayi*. Abbreviations: afn—abfrontal tentacle nerve; bf—brachial fold; cn—cross nerve; cp—coelomic pouch; fg—food groove; fn—frontal tentacle nerve; gfp—groups of frontal perikarya; gu—gutter; it—inner tentacle; lafn—lateroabfrontal tentacle nerve; lbn—lower brachial nerve; lcl—large canal of the lophophoral coelom; ln—lateral tentacle nerve; lol—lamella of loop of the lophophore brachidium; mbn—main brachial nerve; ot—outer tentacle; rbn—radial brachial nerves; sa—second accessory brachial nerve; scl—small canal of the lophophoral coelom.

Each lateral arm is innervated by six brachial nerves: two main, two second accessory, and two lower (Figs. 3B and 6; Suppl. 2). Because each lateral arm is formed by the looped brachial axis, only three nerves belong to each half of the brachial axis: one main, one second accessory, and one lower.

**Figure 7:**
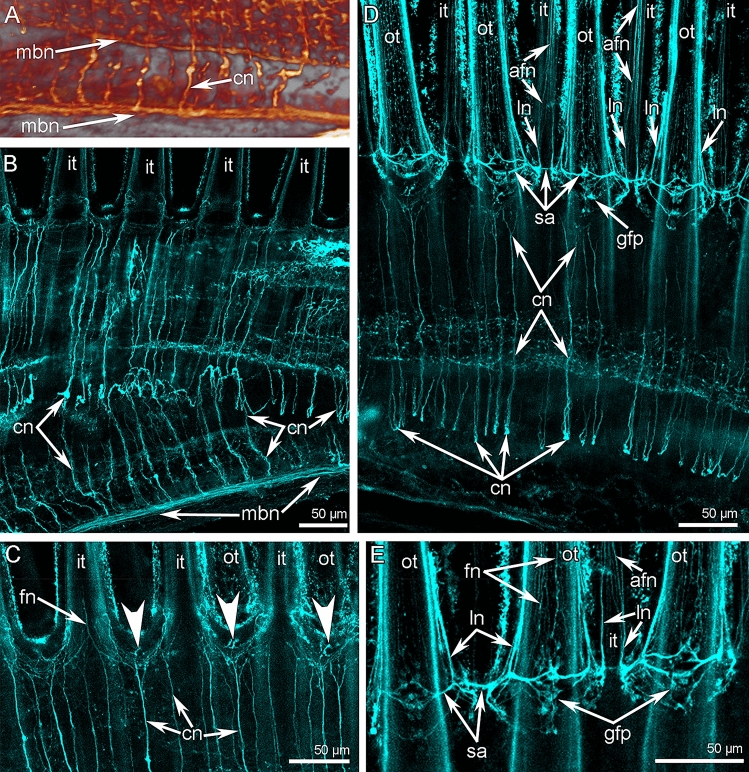
Details of innervation of the lateral arm in *Coptothyris grayi*. Volume rendering (**A**) and Z-projections (**B**–**E**) after immunostaining against acetylated alpha-tubulin (orange—in **A** and cyan in **B**–**E**). (**A**) Two main brachial nerves and cross nerves emanating from them. (**B**) Main brachial nerve and curved cross nerves. (**C**) The most frontal side of the inner tentacles: cross nerves extend to the places between inner tentacles and give rise to two thick short nerves (arrowheads), which extend to the frontal side of the outer tentacles. (**D**) The most frontal side of outer tentacles: groups of perikarya at the base of each tentacle are evident. (**E**) Groups of frontal perikarya and second accessory brachial nerve. Abbreviations: afn—abfrontal tentacle nerve; cn—cross nerve; fn—frontal tentacle nerve; gfp—groups of frontal perikarya; it—inner tentacle; ln—lateral tentacle nerve; mbn—main brachial nerve; ot—outer tentacle; sa—second accessory brachial nerve.

**Figure 8:**
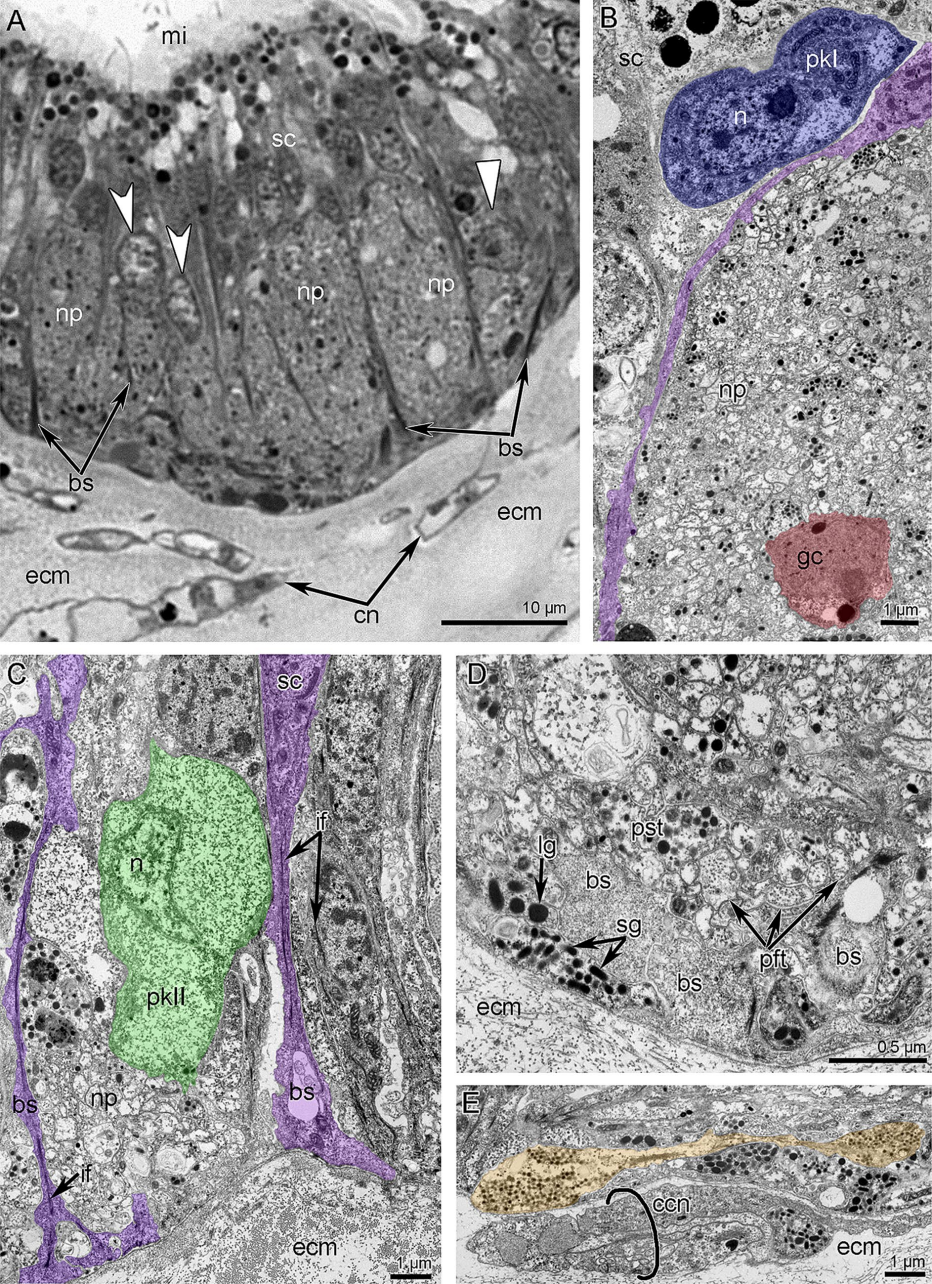
Organization of the main brachial nerve of *Coptothyris grayi*. Semithin (**A**) and ultrathin (**B**–**E**) transverse sections. (**A**) General view of the main brachial nerve: two types of pekikarya (perikaryon of first type is indicated by straight arrowhead; perikarya of second type are pointed by concaved arrowheads) and large cluster of neurites are visible. (**B**) Perikaryon of first type (dark blue) and soma of glial cell (red) between neurites. Basal projection of supportive cell is magenta. (**C**) Perikaryon of second type (green) and numerous basal projections of supportive sells (magenta). (**D**) Cluster of neurites: projections of different types are shown. (**E**) A portion of cluster of neurites containing varicoses of nerve projection of second type (colored). Abbreviations: bs—basal projections of supportive sells; ccn—cells of cross nerve; cn—cross nerve; ecm—extracellular matrix; gc—glial cells; if—intermediate filaments; lg—granules of large diameter; mi—microvilli; n—nucleus; np—cluster of neurites; pft—projections of first type; pkI—perikarya of first type; pkII—perikarya of second type; pst—projections of second type; sc—supportive cells (= cells of radial glia); sg—granules of small diameter.

Two **main brachial nerves** extend along the middle line of each lateral arm and along the middle arm, at the base of the brachial folds (Figs. 2A, 3B, and 6). In each lateral arm, two main brachial nerves are located near each other (Figs. 2B, 3B, and 7A). According to CLSM, each main brachial nerve is formed by longitudinal neurite bundles, which exhibit acetylated alpha-tubulin-like immunoreactivity (Fig. 7B). Each main brachial nerve is completely located in the epithelium of the arm and resembles a compact neurite bundle that is 20–25 µm in diameter (Fig. 8A). Neurite bundles form a large nerve tract, which is divided into several portions by basal projections of supportive cells (= radial glia cells) (Fig. 8A). Transmission electron microscopy (TEM) revealed that these projections contain electron-dense intermediate filaments, which extend from the apical to the basal part of the cells and which adhere to the basal lamina via hemidesmosomes (Fig. 8B, C). Perikarya of at least two types are associated with the nerve (Fig. 8A). Perikarya of the first type have a compact soma with a small nucleus bearing a large nucleolus and electron-dense cytoplasm, which is filled with small mitochondria (Fig. 8B). The perikarya of the second type have a large soma, which contains a large nucleus with electron-light karyoplasm (Fig. 8C). The glial cells are located within the nerve projections (Fig. 8B). These cells have a compact soma and projections, which are filled with large electron-dense granules (Fig. 8D). These projections are numerous between neurites. There are two types of neurites. Neurites of the first type are the most abundant; they have electron-light cytoplasm, which contains many microtubules. Neurites of second type are rare; their cytoplasm is filled with numerous electron-dense granules of different diameter and dense-core vesicles (Fig. 8D); these neurites can form large varicoses, which contain numerous dense-core synaptic vesicles (Fig. 8E).

**Figure 9:**
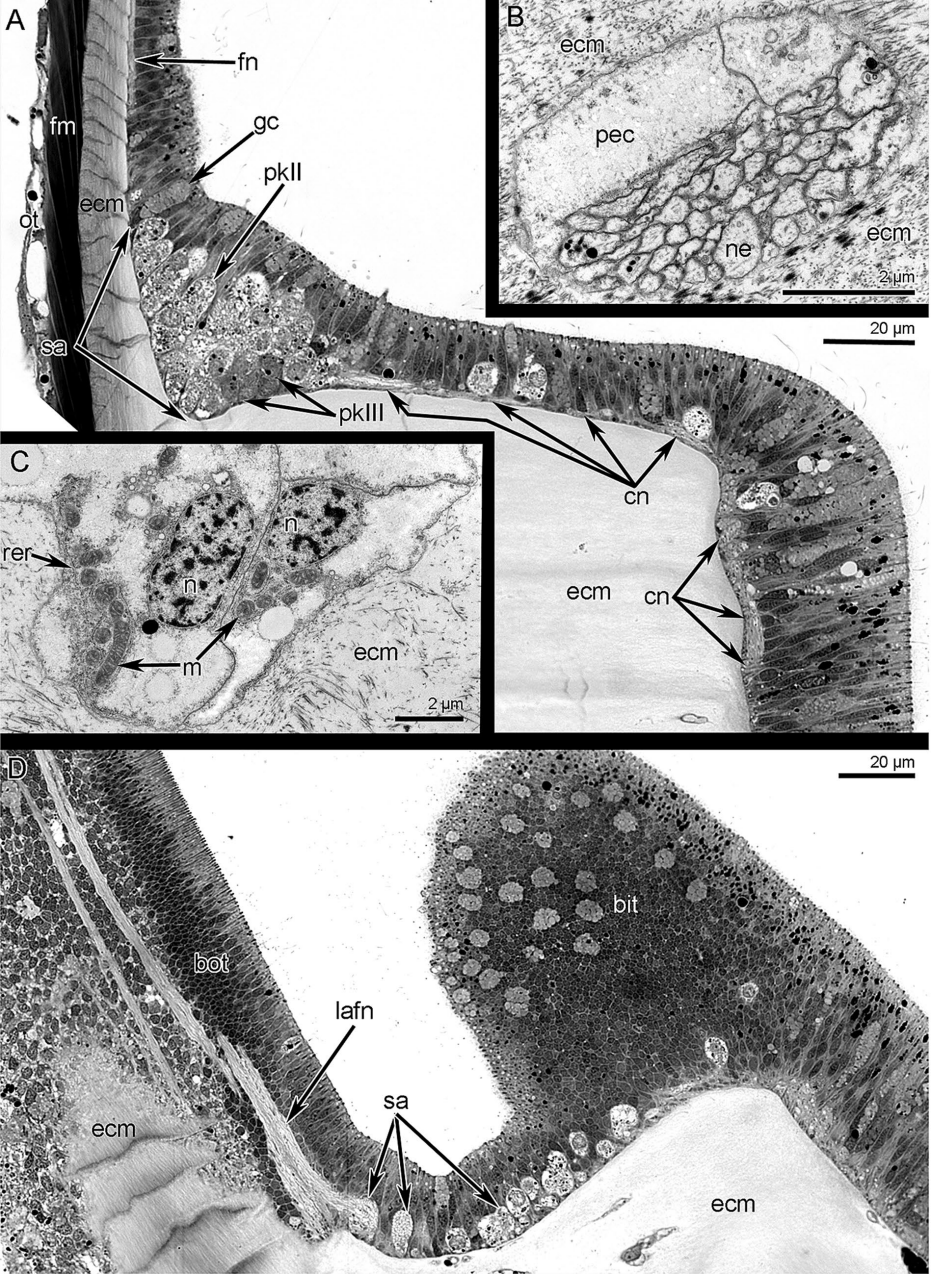
Organization of cross nerves and second accessory brachial nerve of *Coptothyris grayi*. Semithin (**A**, **D**) and ultrathin (**B**, **C**) sections. (**A**) Longitudinal mediofrontal section of the outer tentacle: groups of perikarya of second accessory brachial nerve and cross nerves are visible. (**B**) The transverse section of the cross nerve: neurites and projection of envelop cells are shown. (**C**) Ultrastructure of the envelope cells of cross nerve. (**D**) Longitudinal lateral section of the tentacles base: thick lateroabfrontal tentacle nerve is visible. Abbreviations: bit—base of inner tentacle; bot—base of outer tentacle; cn—cross nerve; ecm—extracellular matrix; fm—frontal tentacle muscle; fn—frontal nerve; gc—gland cell; lafn—lateroabfrontal tentacle nerve; m—mitochondria; n—nucleus; ne—neurite; ot—outer tentacle; pec—projection of envelop cell; pkII—perikarya of second type; pkIII—perikarya of third type; rer—rough endoplasmic reticulum; sa—second accessory brachial nerve.

Each main brachial nerve gives rise to numerous **cross nerves** (Figs. 6 and 7B–D). Each cross nerve penetrates the connective tissue of the brachial arm, extends to the  epithelium of the food groove, and then extends to the base of the tentacles (Fig. 9A). As a consequence, each cross nerve has two ascending parts and one descending part (Figs. 3B, 6, and 7B). The cross nerve is formed by 40–50 neurites of different diameters (Fig. 9B). Some of these neurites contain electron-dense granules and dense-core synaptic vesicles (Fig. 9B). Each cross nerve is associated with several envelop cells, which surround the nerve and have flocculent electron-light cytoplasm with numerous mitochondria, vesicles, and canals of rough endoplasmic reticulum (Fig. 9C).

**Figure 10:**
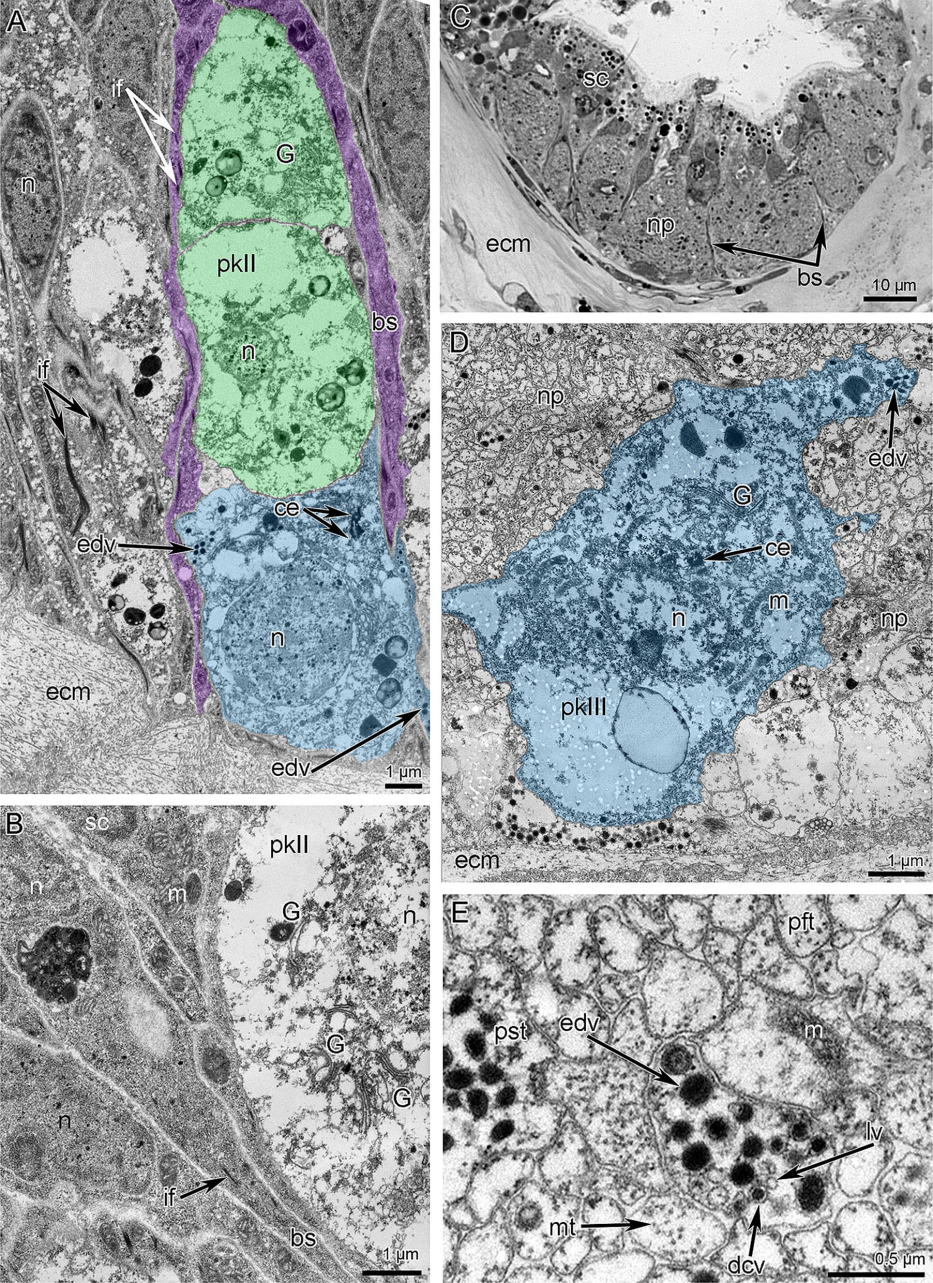
Details of ultrastructure of second accessory (**A**–**B**) and lower (**C-E**) brachial nerves of *Coptothyris grayi*. Ultrathin (**A**–**B**, **D**–**E**) and semithin (**C**) sections. (**A**) A portion of second accessory brachial nerve with perikarya of second type (green) and perikaryon of third type (blue), which are covered by basal projections of supportive cells (magenta). (**B**) A portion of perikaryon of second type: many Golgi apparatuses are visible. (**C**) General view of the lower brachial nerve: large cluster of neurites is crossed by long basal projections of supportive cells (= cells of radial glia). (**D**) A portion of lower nerve with the perikaryoin of third type (blue). (**E**) A portion of cluster of neurites: projections of different types are visible. Abbreviations: bs—basal projection of supportive cells; ce—centriole; dcv—dense-core vesicle; ecm—extracellular matrix; edv—vesicles with electron dense content; G—Golgi apparatus; if—intermediate filaments; lv—synaptic vesicles with electron-lucent content; m—mitochondria; mt—microtubules; n—nucleus, np—cluster of neurites; pft—projections of first type; pkII—perikarya of second type; pkIII—perikarya of third type; pst—projections of second type; sc—supportive cells (= cells of radial glia).

At the base of the tentacles, several cross nerves are grouped together in sites between the inner tentacles and give rise to two short, thick nerves, which extend between the bases of the inner tentacles to the frontal side of the outer tentacles (Fig. 7C). These short nerves are connected to the **second accessory brachial nerve** (Figs. 3B, 6, and 9A, D). This nerve is formed by a group of perikarya and neurites, which are located at the base of frontal side of the outer tentacles and which skirt these tentacles laterally (Figs. 7D, E and 9A, D). These frontal semicircles are connected to each other by bridges, which extend along bases on the abfrontal sides of the inner tentacles (Fig. 7D, E). The ultrastructure of the accessory brachial nerve is similar to that of the main brachial nerve. TEM revealed two types of perikarya in the accessory brachial nerve. One type (i.e., perikarya of the second type—*pkII*), which has a lot in common with perikarya of the main brachial nerve, is the most abundant (Fig. 9A, D). They are large, have electron-light cytoplasm, and contain a large nucleus that lacks a nucleolus (Fig. 10A). Their cytoplasm is filled with numerous Golgi apparatuses and vesicles with electron-lucent content (Fig. 10B). The perikarya of the other type (i.e., perikarya of the third type—*pkIII*) are located at the base of the epithelium and are not abundant (Fig. 9A). These perikarya have electron-dense cytoplasm and contain vesicles with electron-dense content (Fig. 10A). A specific feature of these perikarya is the presence of two centrioles.

**Figure 11:**
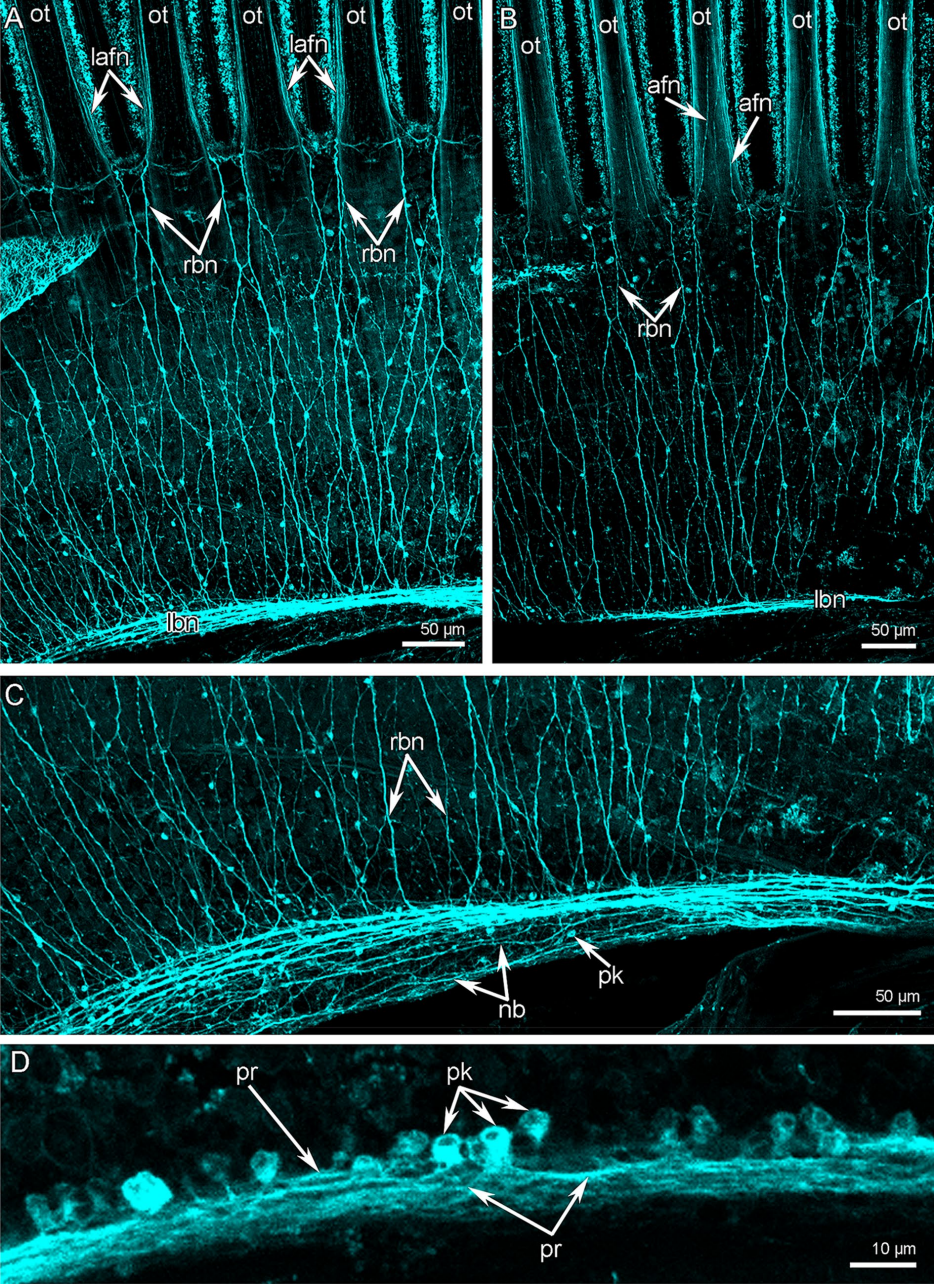
Organization of lower brachial nerve of *Coptothyris grayi.* Z-projections after immunostaining against acetylated alpha-tubulin (cyan). (**A**) A portion of the lateral arm viewed from the outer tentacles. (**B**) The most abfrontal side of outer tentacles. (**C**) A portion of the lower brachial nerve, which gives rise to the radial brachial nerves. (**D**) Magnified portion of the lower brachial nerve: perikarya and their projections are visible. Abbreviations: afn—abfrontal tentacle nerve; lafn—lateroabfrontal tentacle nerve; lbn—lower brachial nerve; nb—neurite bundle; ot—outer tentacle; pk—perikarya; pr—projection; rbn—radial brachial nerves.

The third large brachial nerve is the **lower brachial nerve** (Figs. 2B, 3A, and 6). It extends along both outer sides of each brachial arm and extends about 350 µm from the tentacle base (Fig. 11A). The lower brachial nerve is formed by a thick aggregation of perikarya and their projections (Figs. 10C and 11C).

The large cluster of neurites is penetrated by long and thin basal projection of supportive cells (= radial glia cells) (Fig. 10C). The lower brachial nerve is associated with many perikarya, which form long projections that extend along the nerve (Fig. 11D). According to TEM, there are two types of perikarya, which are identical to the perikarya of the second accessory nerve. In the lower brachial nerve, the basal perikarya have the same peculiarities as perikarya from the second accessory brachial nerve: their cytoplasm contains numerous vesicles with electron-dense content and centrioles (Fig. 10D). These basal perikarya form the projections, which contain vesicles with electron-dense content, dense-core vesicles, and vesicles with electron-light content (Fig. 10E). The lower brachial nerve gives rise to the many radial nerves of the arm. These nerves form a thick nerve net along the outer surface of the brachial arm (Fig. 11A). A pair of radial nerves extend between the lower brachial nerve and the abfrontal side of each outer tentacle (Fig. 11A).

### Innervation of tentacles

Corresponding to their difference in morphology, the outer and inner tentacles differ in innervation. The differences concern the connection of tentacle nerves and brachial nerves and the location of tentacles nerves in the outer and inner tentacles.

The **outer tentacles** contain seven groups of longitudinal nerves: one frontal, two lateral, two latero-abfrontal, and two abfrontal (Fig. 4C). The frontal nerve originates from the groups of perikarya of the second accessory brachial nerve (Figs. 6 and 7E). The frontal nerve is formed by many thin neurite bundles, each of which consists of 5–7 neurites of small diameter (Fig. 5C). These thin neurite bundles are scattered in the epithelium of the frontal grove, and the frontal nerve therefore has weak acetylated alpha-tubulin-like immunoreactivity (Fig. 4C). Lateral tentacle nerves arise from the second accessory nerve (Figs. 6 and 7D, E). Each lateral nerve is formed by three thick neurite bundles, which extended into the epithelium of the lateral ciliated ridges (Fig. 5G). Each neurite bundle consists of 10–20 neurites, which have large diameters and are filled with electron-light cytoplasm (Fig. 5G). Latero-abfrontal nerves originate from the radial nerves of the lophophoral arm, which arise from the lower brachial nerve (Figs. 6 and 11A). Each latero-abfrontal nerve is formed by one or two thick compact neurite bundles, which consist of > 50 neurites (Fig. 5H). The abfrontal zone is innervated by two abfrontal nerves, which originate from the radial nerves of the outer side of the lophophore arm (Figs. 6 and 11B). Accordingly to TEM, abfrontal nerve is very compact group of > 30 neurites, which have electron-lucent cytoplasm and rare synaptic vesicles (Fig. 5D).

In the inner tentacles, there are five tentacle nerves: one frontal, two lateral, and two abfrontal (Fig. 4D). The frontal nerve originates directly from the cross nerves (Figs. 6 and 7C). In one tentacle, the frontal nerve contains neurites from different cross nerves (Fig. 7C). According to immunocytochemistry, each frontal nerve consists of 3–5 separate neurite bundles (Fig. 4D). TEM revealed almost continuous layer of neurites in the basal part of the frontal epithelium (Fig. 5F). All other nerves of the inner tentacles originate from the second accessory nerve (Figs. 6 and 7D). In the inner tentacles, the lateral and abfrontal nerves are organized in the same way as in the outer tentacles except that the inner tentacles lack the latero-abfrontal nerve.

Both outer and inner tentacles are innervated by peritoneal neurites (Fig. 4E). These neurites originate from peritoneal tentacular neurons located at the base of the tentacles (Fig. 4E). Peritoneal neurites extend between the basal lamina and the cells of the coelomic lining (Fig. 5E). They have electron-light cytoplasm and contain bundles of microtubules (Fig. 5E).

## Discussion

### Innervation of the lophophore in brachiopods

The phylum Brachiopoda includes three subphyla: Linguliformea, Craniiformea, and Rhynchonelliformea^24^. Organization of the lophophore nervous system has been studied by different methods in specimens from all three groups, including the following species: *N. anomala*^23,25^, *Discinisca lamellosa*^26^, *L. anatina*^4^, *Gryphus vitreus*^27^, and *H. psittacea*^22^. Innervation of the lophophore in brachiopods has mostly been studied via light microscopy^25–27^, and only four species have been studied with TEM, immunocytochemistry, and CLSM^4,22,23,28^. According to all data, the central nervous system of rhynchonelliformean brachiopods includes two ganglia, the subenteric and the supraenteric, which are located under and above the mouth, respectively. Adult brachiopods from the Linguliformea and Craniiformea lack the supraenteric ganglion^25,26^. Juveniles of *N. anomala* from the Craniiformea, however, have the supraenteric ganglion^23^.

The subenteric ganglion gives rise to the lower brachial nerve, whereas the supraenteric ganglion gives rise to the main brachial nerve. The main brachial nerve is connected to the accessory brachial nerve via numerous cross nerves, which extend into the connective tissue of the lophophore arms. These three brachial nerves, i.e., the main, accessory, and lower, are the major nerves of the lophophore in all brachiopods studied to date^4,22,23,25–27^. Two recent studies of lophophore innervation have revealed the presence of a second accessory nerve in the rhynchonelliform *H. psittacea*^22^ and in the juveniles of craniiform *N. anomala*^23^. Parts of this brachial nerve are represented by groups of FMRF-amide-like immunoreactive perikarya and have been previously described in *L. anatina*^4^. Brachiopods therefore have four major brachial nerves: the main, accessory, second accessory, and lower.

In *G. vitreus*, which has the plectolophe (the most complex type of lophophore among recent brachiopods), only the main and lower brachial nerves have been reported in the lateral arms^27^. The study also described the accessory brachial nerve in the medial arm of the *G. vitreus* lophophore.

According to our data, the plectolophe of *C. grayi* is innervated by three brachial nerves: the main, second accessory, and lower; the accessory brachial nerve is completely absent. In *H. psittacea*, the accessory brachial nerve is present but does not contribute to the innervation of the tentacles: the cross nerves do not merge with the accessory brachial nerve but skirt it and extend to the second accessory nerve^22^. This state may be regarded as the first step in the reduction of the accessory nerve in the brachiopods from the Rhynchonelliformea, which is the most morphologically and taxonomically diverse group of brachiopods (see below).

### Innervation of tentacles

In brachiopods, all tentacles are highly specialized. The specialization is expressed by the presence of epithelial zones, which extend along different sides of the tentacle and which are associated with longitudinal nerves and muscle bundles^29^. All other lophophorates have a similar organization of tentacles^23,30^, but brachiopod tentacles are more specialized than phoronid tentacles. This specialization is also manifested by double row of tentacles and the formation of prominent lateral epithelial ridges in brachiopod tentacles. The innervation differs in the inner and outer tentacles, i.e., the outer tentacles but not the inner tentacles have lateroabfrontal nerves. The lateroabfrontal tentacle nerves have also been documented in the outer tentacles of *H. psittacea*^22^. The presence of these nerves may be associated with the great extension of the lateroabfrontal zone in the outer tentacles. The lateroabfrontal zone also contains many gland cells, which produce the mucous that facilitates the “peeling off” of waste particles from the lophophore^31^. The peeling off involves a reversal in the beating of cilia, which may also require the additional innervation provided by the lateroabfrontal tentacle nerves^31^.

### Ultrastructure of the lophophore nerves

In all brachiopods studied to date, the lophophoral nerves are located in the lophophore epithelium^4,22,28^. This epithelium is formed by columnar cells with long, thin basal projections, which contain electron-dense filaments and that are attached to the basal lamina via hemidesmosomes. This organization of epithelial cells, which are associated with the nerve tissues, suggests that these epithelial cells are “radial glia”^28,32,33^. These epithelial cells protect the nerve cells and supply them with nutrients. In other brachiopods and some other lophophorates, typical glial cells have been described^4,34^. These cells are located within the cluster of neurites and form projections, which contain large electro-dense granules and surround neurite bundles and perikarya. Such glial cells were found in the main brachial nerve of *C. grayi* in the current study.

Two kinds of perikarya, i.e., large perikarya with electron-lucent cytoplasm (perikarya of second type) and small perikarya with electron dense-cytoplasm, (perikarya of first and third types), have been discovered in the lophophoral nerves of *C. grayi*. Both kinds of perikarya form projections of two different types, which can be detected in all brachial nerves. The same perikarya were recently described in the supraenteric and subenteric ganglia of *C. grayi*^28^. The main brachial nerve of *L. anatina* contains at least five types of perikarya^4^. The small number of perikarya types in *C. grayi* and in *H. psittacea*^22^ may correlate with the general reduction of the main brachial nerve (see below).

Perikarya of different types apparently promote different functions. Without special physiological studies it is difficult to suggest exactly function of certain types of nerve cells. Usually, large perikarya with electron-lucent cytoplasm are regarded as motor neurons^34^. Accordingly to their relatively large diameter and presence of electron lucent cytoplasm, perikarya of second type of *C. grayi* may be regarded as cell bodies of motor neurons. At the same time, perikarya of second type contain many Golgi apparatuses and probably are able to produce neurosecretory substances. In *C. grayi*, perikarya of first and third types have similar organization and contain centrioles and probably originate from ciliated sensory cells, which lost a cilium. Centrioles have been also described in perikaria of phoronids^34^. Perikarya of first and third types can be regarded as cell bodies of intercalary or sensory neurons. The presence of perikarya of different types in all brachial nerves of *C. grayi* allows to suggest the similar function for all these nerves. Combination of different function in one nerve probably reflects general simplicity of the nervous system of brachiopods^22^.

### Modification of the lophophore nervous system in brachiopods

Our new results and previously published data indicate that the lophophore nervous system in recent brachiopods generally consists of four major brachial nerves: the main, accessory, second accessory, and lower. According to immunocytochemistry and CLSM, this general plan of the nervous system differs between the Linguliformea and Rhynchonelliformea^4,22^.

**Figure 12:**
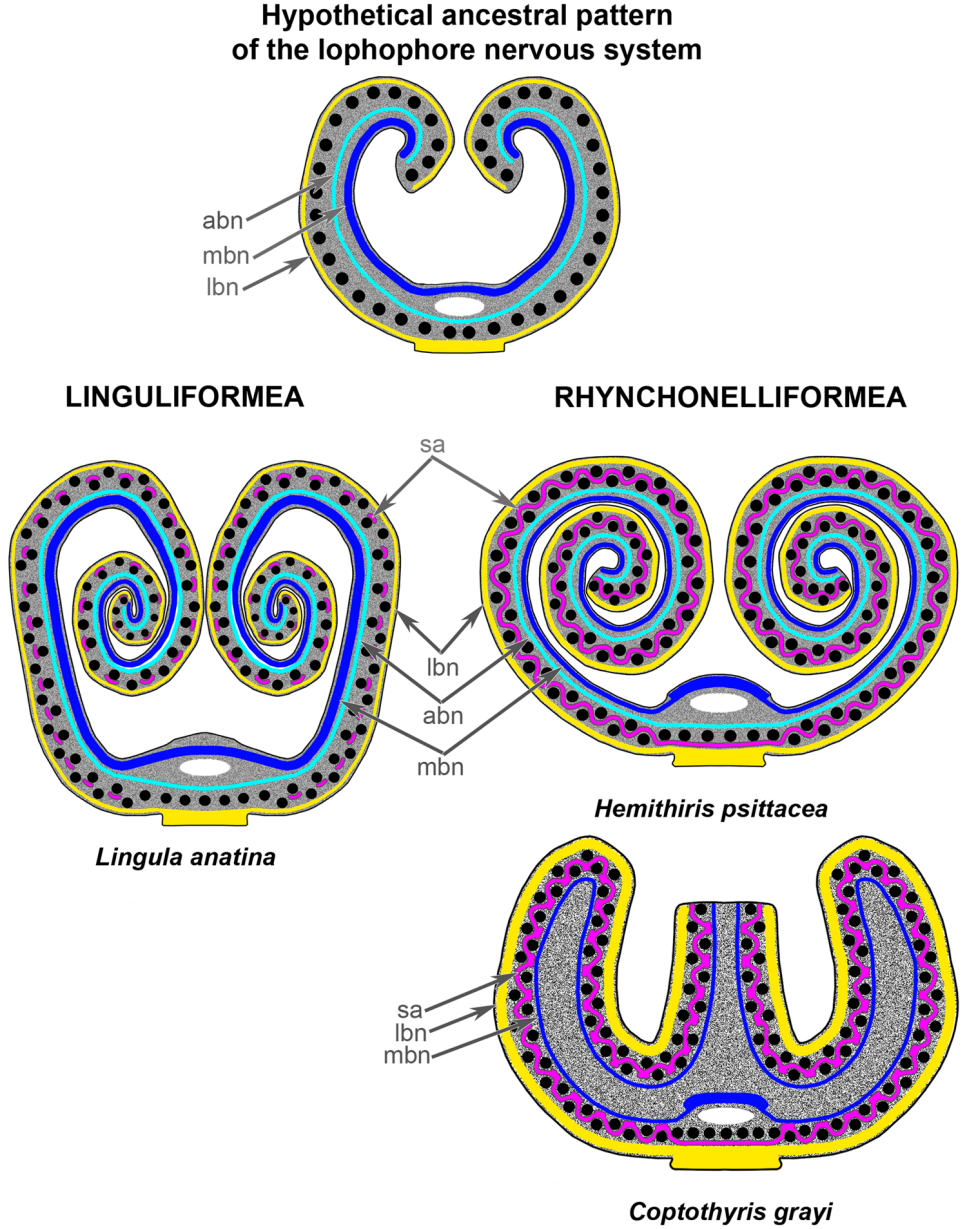
Scheme of the lophophore nervous system in various brachiopods. The black circles indicate tentacles. Abbreviations: abn—accessory brachial nerve; lbn—lower brachial nerve; mbn—main brachial nerve; sa—second accessory brachial nerve.

In linguliformes, e.g., *L. anatina*, the main and accessory brachial nerves are prominent and contribute substantially to the innervation of the tentacles (Fig. 12). The main brachial nerve in *L. anatina* contains five types of perikarya and is formed by numerous neurite bundles including the giant nerve fibers^4^. The lower brachial nerve, in contrast to the main brachial nerve, is weakly pronounced and is formed by several thin neurite bundles, which are not grouped together. *L. anatina* also has a row of separated groups of perikarya, which are located at the base of tentacles, between them (Fig. 12). The location of this row is similar to the location of the second accessory nerve of *H. psittacea* and *C. grayi*.

Although some rhynchonelliforms, e.g., *H. psittacea*, have all four brachial nerves, rhynchonelliforms differ from linguliforms in that the accessory brachial nerve does not connect with the cross nerves and does not contribute to tentacle innervation^22^. In the rhynchonelliform *C. grayi*, the accessory brachial nerve is absent. Interestingly, rhynchonelliforms have only half the number of perikarya in brachial nerves as the linguliform *L. anatina*.

The presence of a second accessory brachial nerve appears to be an advanced state in brachiopods and has not been reported in other lophophorates^6^. The presence of this nerve is correlated with the appearance of a double row of tentacles, which include alternating outer and inner tentacles. The appearance of alternating tentacles results in a pattern of tentacle innervation that is not typical for lophophorates; most lophophorates do not have alternating tentacles, and innervation of adjacent tentacles for most lophophorates is provided by intertentacular nerves^6,18^. The latter innervation helps coordinate the functioning of adjacent tentacles. A correlation between the formation of a double row of tentacles and the appearance of the second accessory brachial nerve has been clearly shown in juveniles of *N. anomala*^23^.

The comparison of the size and complexity of the second accessory nerve in *L. anatina*, *H. psittacea*, and *C. grayi* may inform evolution of the lophophore innervation in brachiopods. The increased complexity of the secondary accessory nerve is correlated with an increased complexity of the brachial axis and of lophophore morphology. Thus, *L. anatina* lacks a sophisticated spirolophe with several coils^12^, and its second accessory brachial nerve is represented by separated nerve nodes^4^; *H. psittacea* has a very complex spirolophe with many coils and a prominent second accessory brachial nerve^22^; and *C. grayi* has the most complex type of lophophore, the plectolophe, and the most pronounced second accessory nerve, which includes large groups of frontal perikarya (Fig. 12).

In all brachiopods studied to date, the supraenteric ganglion (or the main brachial nerve if that ganglion is absent) and the subenteric ganglion are connected to each other via circumenteric connectives^25–28^. As shown in the current study, these connectives are present in *C. grayi*. In addition to circumenteric connectives, *C. grayi* has thick nerves that extend from both ganglia to the nerves of the median brachial arm. The presence of these nerves may be explained by the complexity of the lophophore morphology: each arm must be innervated directly from the central nervous system to facilitate a rapid and proper response to stimuli. No other studied brachiopod developed (Fig. 12).

Has these additional nerves, probably because the lophophore morphology of other brachiopods is simpler than that of *C. grayi*, which has a plectolophe.

As a result, the nervous system of the plectolophe exhibits both simplified and advanced features. Thus, the absence of an accessory nerve can be regarded as a simplification, which may correlate with the reduction of the brachial muscle that accompanied the development of supporting skeletal structures^35^. The presence of a second accessory nerve and of several additional nerves extending into the medial arm is apparently correlated with the very complex morphology of the plectolophe.

## Conclusion

Because the earliest extinct brachiopods had a simple spirolophe with a single row of tentacles^36–38^, we infer that the ancestral pattern of the nervous system of the brachiopod lophophore did not include a second accessory nerve. We suggest that the ancient lophophore of all lophophorates had many different muscles^17^. In “inarticulate” brachiopods (i.e., linguliforms and craniiforms), a system of well-developed muscles works as supportive structures of the lophophore^35^, and the presence of such well-developed muscles requires the presence of many different brachial nerves. In “articulate” brachiopods (i.e., rhynchonelliforms), which provide skeletal support of the lophophore^14^, the lophophoral muscles are extremely reduced^35^; that reduction in muscle development led to a reduction of some brachial nerves, i.e., the absence of the accessory brachial nerve and the diminution of the main brachial nerve. According to this hypothetical scenario, the lophophore nervous system in brachiopods evolved from the presence of three brachial nerves (i.e., main, accessory, and lower), to the appearance of four brachial nerves due to formation of a double row of tentacles, and then to reduction of some nerves (including the accessory brachial nerve) because of the reduction of muscles and to the intensification of other nerves (the second accessory brachial nerve) due to the increased complexity of the lophophore morphology.

## Materials and methods

### Animals

Adults of *Coptothyris grayi* (Davidson, 1852) were collected in July 2015 in Vostok Bay, Sea of Japan. The collected specimens were relaxed in 7% MgCl_2_ for 20 min and then photographed using a Leica M165C stereomicroscope equipped with a Leica DFC420 digital camera (Leica Microsystems GmbH, Wetzlar, Germany). Specimens were dissected to obtain the lophophore. Parts of the lophophores were fixed for semi-thin sectioning, scanning electron microscopy (SEM), TEM, immunocytochemistry, and CLSM.

### Microscopy

For SEM and TEM, lophophores were fixed in 2.5% glutaraldehyde in 0.05 M cacodylate buffer containing NaCl and were then post-fixed in 1% osmium tetroxide in the same buffer. For SEM, parts of the lophophores were dehydrated in ethanol followed by an acetone series, critical point dried, and then sputter coated with platinum-palladium alloy. Specimens were examined with a JEOL JSM-6380LA scanning electron microscope (JEOL Ltd., Tokyo, Japan).

For semi-thin sectioning and TEM, specimens were dehydrated in ethanol and embedded in Embed-812 resin (Electron Microscopy Science, USA). Semi-thin and thin sections were prepared with a Leica UC7 ultramicrotome (Leica Microsystems GmbH, Wetzlar, Germany). Semi-thin sections were stained with methylene blue, observed with a Zeiss Axioplan2 microscope, and photographed with an AxioCam HRm camera (Carl Zeiss, Oberkochen, Germany). Ultrathin sections were stained with uranyl acetate (0.5%) and lead citrate (0.4%) and then examined with a JEOL JEM 100B electron microscope (JEOL Ltd., Tokyo, Japan).

For immunocytochemistry, lophophores of *C. grayi* were fixed in a 4% paraformaldehyde solution and washed in phosphate buffer (pH 7.4) (Fisher Scientific, Pittsburgh, PA, USA) with Triton X-100 (10%) (Fisher Scientific) (PBT). Non-specific binding sites were blocked with 10% normal donkey serum (Jackson ImmunoResearch, Newmarket, Suffolk, UK) in PBT. The specimens were incubated in primary antibody (mouse anti-ɑ-Tubulin; 1:700; cat. # 32-2500; TermoFisher Scientific) in phosphate buffer with Triton X-100), washed in PBT, exposed to the secondary antibody (goat anti-mouse conjugated with Alexa-635; 1:1000; cat. # A-31574; TermoFisher Scientific), washed in phosphate buffer, embedded in Murray Clear, mounted on a glass slides covered with poly-L-lysine (Sigma-Aldrich, St. Louis, MO, USA), and examined with a Nikon Eclipse Ti confocal microscope (Moscow State University, Moscow, Russia). Z-projections were prepared using Image J version 1.43 software. Volume renderings were prepared using Amira version 5.2.2 software (Thermo Fisher Scientific, MA, USA). TEM micrographs and Z-projections were processed in Adobe Photoshop CS3 (Adobe World Headquarters, San Jose, California, USA) to prepare panoramas and combinations of Z-projections.

### Ethics approval and consent to participate

The use of brachiopods in the laboratory does not raise any ethical issues, and therefore approval from regional and local research ethics committees was not required. The field sampling did not involve endangered or protected species. In accordance with local guidelines, permission for collection of material was not required.

### Consent for publication

The authors have read the manuscript and consent to its publication.

## Supplementary Information

Supplementary Information 1.

Supplementary Information 2.

Supplementary Information 3.

## Data Availability

The data sets analyzed during the current study are available from the corresponding author in response to reasonable requests.
