# Supplementary figures and images for: The nervous system of the most complex lophophore provides new insights into the evolution of Brachiopoda
Source: Sci Rep. 2021 Aug 10;11:16192. doi: 10.1038/s41598-021-95584-5 (PMC8355163; doi:10.1038/s41598-021-95584-5)

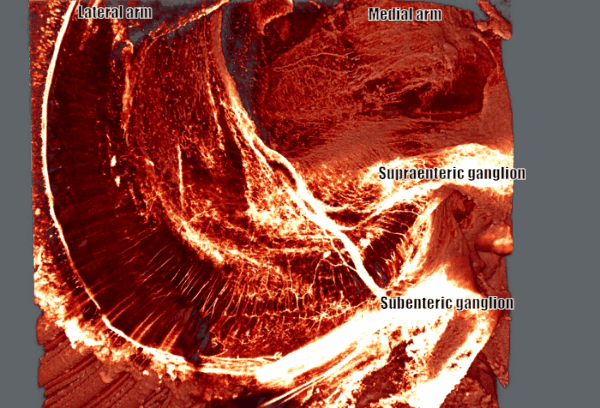

Supplement: Supplementary file 1 — Supplementary Information 1. [file 41598_2021_95584_MOESM1_ESM.gif]

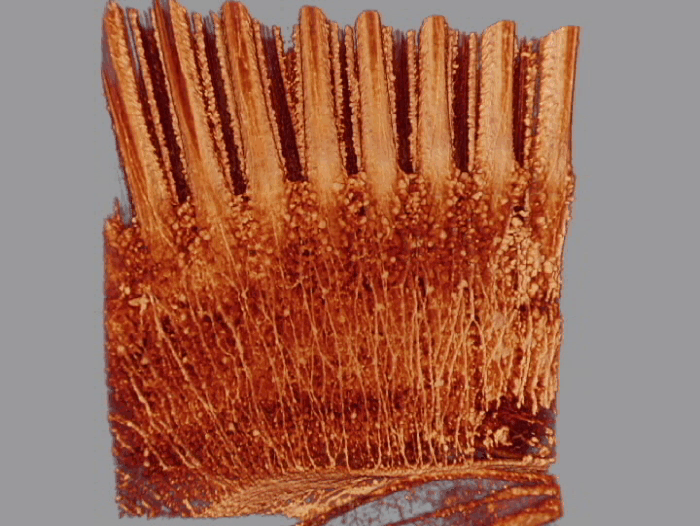

Supplement: Supplementary file 2 — Supplementary Information 2. [file 41598_2021_95584_MOESM2_ESM.gif]
