# Supplementary material for: The nervous system of the most complex lophophore provides new insights into the evolution of Brachiopoda
Source: Sci Rep. 2021 Aug 10;11:16192. doi: 10.1038/s41598-021-95584-5 (PMC8355163; doi:10.1038/s41598-021-95584-5)
Supplement: Supplementary file 3 — Supplementary Information 3. [file 41598_2021_95584_MOESM3_ESM.docx]

**Supplementary Figure Legends**

**Supplement material 1.** Running-Z projection of central portion of the lophophore with parts of lateral and median arms; volume rendering after immunostaining against acetylated alpha-tubulin; CLSM.

**Supplement material 2.** Running-Z projection of a portion of lateral brachial arm from the most outer lower brachial nerve to the inner tentacles. Volume rendering after immunostaining against acetylated alpha-tubulin; CLSM.
